# TOMM34 serves as a candidate therapeutic target associated with immune cell infiltration in colon cancer

**DOI:** 10.3389/fonc.2023.947364

**Published:** 2023-02-09

**Authors:** Zhigui Li, Hongzhao Yang, Jianbo Liu, Li Li, Xiaodong Wang

**Affiliations:** ^1^ Department of Gastrointestinal Surgery, West China Hospital, Sichuan University, Chengdu, China; ^2^ West China School of Medicine, Sichuan University, Chengdu, China

**Keywords:** Tomm34, colon cancer, prognostic biomarker, immune infiltration, immunotherapy

## Abstract

**Background:**

Colon cancer represents one of the most pervasive digestive malignancies worldwide. Translocase of the outer mitochondrial membrane 34 (TOMM34) is considered an oncogene and is implicated in tumor proliferation. However, the correlation between TOMM34 and immune cell infiltration in colon cancer has not been investigated.

**Materials and methods:**

Based on multiple open online databases, we performed integrated bioinformatics analysis of TOMM34 to evaluate the prognostic value of TOMM34 and its correlation with immune cell infiltration.

**Results:**

TOMM34 gene and protein expression levels were elevated in tumor tissues compared with normal tissues. Survival analysis revealed that upregulation of TOMM34 was significantly associated with poorer survival time in colon cancer. High TOMM34 expression was dramatically related to low levels of B cells, CD8+ T cells, neutrophils, dendritic cells, PD-1, PD-L1 and CTLA-4.

**Conclusions:**

Our results confirmed that high expression of TOMM34 in tumor tissue correlates with immune cell infiltration and worse prognosis in colon cancer patients. TOMM34 may serve as a potential prognostic biomarker for colon cancer diagnosis and prognosis prediction.

## Background

Colon cancer is one of the most pervasive digestive malignancies worldwide (1, 2). It is estimated that there were 104,610 new cases and 53,200 deaths in America in 2020. In China, the incidence and mortality of colon cancer have increased recently (2). To date, colon cancer has become a heavy public health burden worldwide. Despite improvements in precise diagnosis and combined therapy, colon cancer remains the main cause of cancer-related mortality around the world (3). Therefore, it is imperative to identify novel prognostic biomarkers and promising therapeutic targets against colon cancer.

Mitochondria are essential organelles that are indispensable to the viability of eukaryotic cells and play crucial roles in metabolism, bioenergetics and signaling. Mitochondrial dysfunction is a hallmark of cancer that leads to aberrant mitochondrial functions, such as integration of metabolism, ATP production, regulation of apoptosis and calcium homeostasis (4). Previous studies have shown that tumor cells can spoil respiration and elevate lactate production, a phenomenon referred to as impeded oxidative phosphorylation and increased aerobic glycolysis, also known as the Warburg effect (5). Owing to rapid proliferation, tumor cells exhibit a high degree of protein synthesis and the absence of essential nutrients and oxygen. To overcome these difficulties, tumor cells increase the components of the protein chaperone system, including Hsp70 and Hsp90 (6, 7). The abnormal mitochondrial metabolism of tumor cells attenuates the proliferation and function of tumor-infiltrating lymphocytes, thereby leading to immune evasion and immunosuppression (8).

The translocase of the outer mitochondrial membrane (TOM) complex plays an important role in the import of the preprotein synthesized in the cytoplasm into the mitochondria. Mitochondria are bound by double-layer membranes, including the inner membrane, intermembrane space and outer membrane (9, 10). Comprehensive genetic analysis has revealed that translocase of the outer mitochondrial membrane 34 (TOMM34) is considered an oncogene and is implicated in tumor proliferation. A previous study revealed that Hsp70 and Hsp90 play pivotal roles in mitochondrial protein folding and stabilization (11). The interactions between chaperones and cochaperones could affect chaperone activities (12). TOMM34 is a cochaperone of Hsp70 and Hsp90 in mitochondrial protein import. Previous studies have revealed that TOMM34 is implicated in mitochondrial protein processing (13, 14). Subsequently, further studies confirmed that TOMM34 interacts with Hsp70 and Hsp90 and mediates protein folding (15–17).

Investigation of cancer antigens may result in the development of promising and effective anticancer drugs (18, 19). Several clinical trials have investigated the safety and immunological response of peptide vaccines derived from the tumor-associated protein TOMM34 (20–22). In addition to the ovaries and testes, TOMM34 is overexpressed in tumor tissues, such as colorectal cancer, early invasive breast cancer, bladder cancer, lung cancer and hepatocellular carcinoma (13, 18, 23, 24). TOMM34 is expressed in the cytoplasm and nucleus of tumor cells. Dendritic cells are antigen-presenting cells that present tumor antigens to lymphocytes and induce cytotoxic T-lymphocytes (CTLs) *in vivo*. The induced CTLs have the ability to identify the tumor antigen and to show an anticancer effect on tumor cells overexpressing the tumor antigen. A recent study indicated that overexpression of TOMM34 was remarkably related to high stage, muscle invasion, high grade and shorter survival in urothelial carcinoma of the bladder (25).

These findings suggest an underlying relationship between TOMM34 and tumorigenesis as well as immunotherapy. The aim of the study was to understand the functional roles and to unearth the potential diagnostic and prognostic value of TOMM34 in colon cancer. In the present study, we analyzed the gene expression, protein expression and gene alterations of TOMM34 in colon cancer from several comprehensive databases. Moreover, we also analyzed the correlation between TOMM34 and immune cell infiltration in the tumor microenvironment.

## Materials and methods

### Data acquisition and processing

Gene expression profiles were downloaded from The Cancer Genome Atlas (TCGA) colon adenocarcinoma (COAD) database (https://portal.gdc.cancer.gov). Under the threshold of log2FC=1.5 and Padj=0.05, the R package “edgeR” was utilized to evaluate the differentially expressed genes (DEGs) for further analysis. Mutation profiles were obtained from the UCSC Xena project (http://xena.ucsc.edu).

### UALCAN

UALCAN (http://ualcan.path.uab.edu/) is a comprehensive and interactive online tool for analyzing cancer OMICS data (26). We evaluated the relationship between TOMM34 expression and distinct clinicopathological traits (race, sex, age, weight, stage, histology, nodal metastasis status, TP53 mutation status) in the “TCGA” and “CPTAC” modules of UALCAN.

### Gene expression profiling interactive analysis (GEPIA)

We utilized the GEPIA online tool (http://gepia.cancer-pku.cn/index.html ) to perform gene expression analysis. The expression of TOMM34 between tumor tissues and normal tissues was investigated with the option of matching TCGA normal and GTEx data in the “Expression DIY” module of GEPIA. Survival analysis of TOMM34 was performed in the “survival plots” module.

### Analysis of the interaction of TOMM34-related genes and proteins

We employed the GeneMANIA database (http://www.genemania.org) to construct the TOMM34 interaction network. The protein−protein interaction (PPI) network of TOMM34 was constructed by using the STRING online tool (https://string-db.org, version 11.5). The main parameters were as follows: protein name (TOMM34), organism (Homo sapiens), max number of interactors to show (no more than 20 interactors), and minimum required interaction score (medium confidence 0.400).

### Gene set enrichment analysis (GSEA), gene ontology (GO) and Kyoto encyclopedia of gene and genomes (KEGG) analysis

All patients were divided into low and high groups on the basis of TOMM34 expression. GSEA was employed to explore the potential mechanisms of TOMM34. Data mining from TCGA database was employed to recognize the positively (2155 genes) and negatively (363 genes) coexpressed genes. These coexpressed genes were utilized to perform GO enrichment and KEGG pathway analyses to investigate the biological functions of TOMM34 in colon cancer. GO enrichment analyses included biological processes (BPs), cellular components (CCs) and molecular functions (MFs).

### cBioPortal

Genetic alteration analysis was employed by using cBioPortal (http://www.cbioportal.org/) to obtain TOMM34 gene data. We chose two colon cancer datasets with 693 patients for further analysis. We utilized the “Cancer Types Summary” module of cBioPortal to analyze the genomic alteration types and alteration frequency of TOMM34 in colon cancer.

### Mutation analysis

Based on the expression of TOMM34, all patients were divided into low and high TOMM34 expression groups. The R package “maftools” was utilized to perform tumor mutation analysis. Tumor mutation burden (TMB) among the low and high TOMM34 groups was visualized through the R package “maftools”.

### Tumor immune estimation resource (TIMER)

TIMER (https://cistrome.shinyapps.io/timer/) is a comprehensive web portal for systematic analysis of immune cell infiltration in a variety of malignancies. We assessed TOMM34 expression in a variety of malignancies through the module “Diff Exp” of TIMER. By using the TCGA-COAD dataset, the relationship between TOMM34 and immune cell infiltration levels (dendritic cells, CD4+ T cells, CD8+ T cells, B cells, neutrophils and macrophages) was evaluated in the “gene” module. The correlation of TOMM34 expression and distinct immune checkpoints (PD-1, PD-L1 and CTLA-4) was analyzed in the “correlation” module of TIMER.

### Single gene set enrichment analysis (ssGSEA)

We performed ssGSEA to evaluate the content of 27 immune cell types in the tumor microenvironment based on the validated gene signature matrix (**Supplementary Table S1**). The correlations between TOMM34 expression and the immune cell subpopulation were evaluated in colon cancer.

### Tissue microarray and immunohistochemistry

Immunohistochemistry was used for semiquantitative analysis of TOMM34 expression. All tumor samples obtained from the West China Hospital Biobank of Sichuan University were made into donor paraffin blocks. We first evaluated paraffin sections by hematoxylin and eosin staining and marked representative tissue regions in donor paraffin blocks. Then, using a specialized manual tissue arranger, cylinders with a diameter of 1.5 mm were selected from these marked areas and placed into empty paraffin blocks. Immunohistochemistry of tissue microarrays was performed as follows: 4-mm-thick paraffin sections were deparaffinized in xylene, rehydrated, and washed three times in phosphate buffered saline (PBS) for 10 min each. Antigen retrieval was performed in an autoclave for 3 min, and endogenous peroxidase activity was inhibited using a 3% hydrogen peroxide solution, followed by incubation of sections with primary antibodies overnight at 4°C. Subsequently, sections were rinsed thoroughly with PBS and incubated with secondary antibodies. PBS was removed, 3,3’-diaminobenzidine tetrahydrochloride was added dropwise for color development, and the immunohistochemical reaction was observed. Finally, the samples were counterstained with hematoxylin, washed with water, dehydrated with graded alcohol, cleared with xylene, and sealed with neutral gum.

### Morphometric assay

An image analysis system (Olympus upright microscope-BX53, Japan) was utilized. Immunohistochemical staining was observed at 20x magnification, and then the expression of TOMM34 was evaluated and counted. We finally screened 206 samples from 155 patients in this study.

### Liquid chromatography−mass spectrometry/mass spectrometry (LC−MS/MS) analysis

LC−MS/MS was used for quantitative analysis of TOMM34 expression. All samples were collected at West China Hospital with the approval of the research ethics committee. LC−MS/MS analysis was performed with a TMT-based isobaric labeling strategy. Sample processing included protein extraction and digestion, isobaric labeling of TMT 10plex reagents (Thermo Scientific), peptide fractionation by high-performance liquid chromatography (HPLC), and desalting and loading of the sample onto a trap column and an analytical column for LC−MS/MS analysis. LC−MS/MS analysis was performed by an EASY-nLC 1000 nanoflow LC instrument coupled to a Q Exactive Plus Quadrupole-Orbitrap mass spectrometer (Thermo Fisher Scientific).

### Statistical analysis

With hazard ratios (HRs) and Cox *P* values from a log-rank test, the results of Kaplan−Meier plots are shown. Spearman’s correlation analysis was employed to investigate the correlations between TOMM34 and immune cell types. A two-sided *P* value<0.05 was considered statistically significant.

## Results

### TOMM34 expression analysis

**Figure 1:**
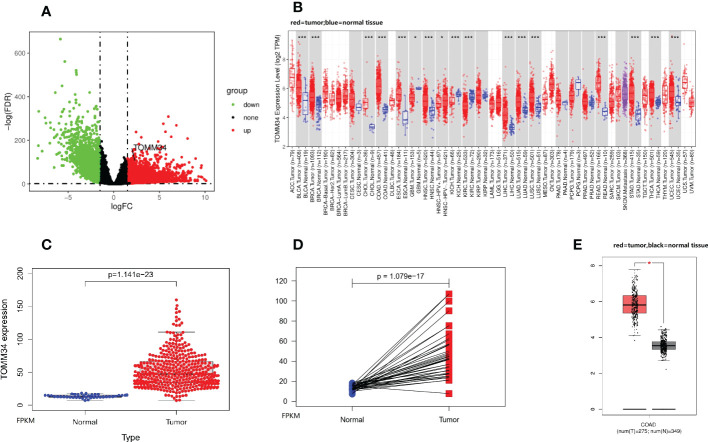
TOMM34 expression analysis in colon cancer. **(A)** Volcano plot showing gene expression. **(B)** TOMM34 expression in divergent types of cancer, indicating that TOMM34 is highly expressed in a variety of cancers. **(C)** TOMM34 expression was elevated in tumor tissues compared with normal tissues obtained from the TCGA-COAD dataset, suggesting that the expression of TOMM34 was increased in tumor tissue. **(D)** TOMM34 expression in tumor tissues and paired normal tissues obtained from the TCGA-COAD dataset, suggesting that the expression of TOMM34 was increased in tumor tissue. **(E)** TOMM34 expression in tumor tissues and normal tissues obtained from the GEPIA database, suggesting high expression of TOMM34 in tumor tissue. *p<0.05.

In the present study, we identified 1877 DEGs (1070 upregulated and 807 downregulated) from the TCGA-COAD dataset (**Figure 1A**). Apart from glioblastoma multiforme, kidney chromophobe and kidney renal clear cell carcinoma, analysis using the TIMER database revealed that TOMM34 expression was elevated in 13 TCGA tumors compared with normal tissues, including bladder urothelial carcinoma, breast invasive carcinoma, cholangiocarcinoma, colon cancer, esophageal carcinoma, head and neck squamous cell carcinoma, liver hepatocellular carcinoma, lung adenocarcinoma, lung squamous cell carcinoma, rectum adenocarcinoma, stomach adenocarcinoma, thyroid carcinoma and uterine corpus endometrial carcinoma (**Figure 1B**). In particular, overexpression of TOMM34 was observed in colon cancer compared with normal tissues (**Figures 1C-E**), suggesting that TOMM34 may play a crucial role in the pathogenesis of colon cancer.

### Correlations between TOMM34 expression and clinical traits in colon cancer patients

**Figure 2:**
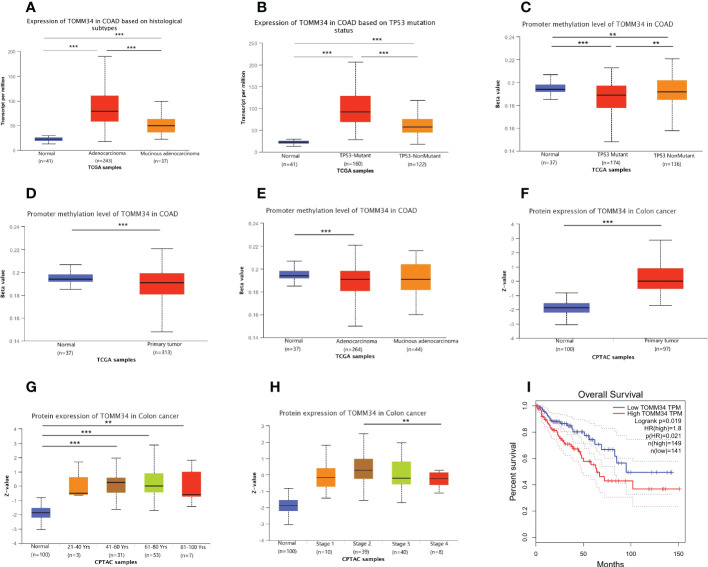
Correlation between TOMM34 expression and clinicopathological traits obtained from TCGA and CPTAC samples. **(A)** Histological subtypes from TCGA samples, indicating that TOMM34 expression was associated with histological subtype. **(B)** TP53 mutation status from TCGA samples, indicating that TOMM34 expression was associated with TP53 mutation status. **(C)** Promoter methylation level in distinct TP53 mutation statuses from TCGA samples, suggesting that TOMM34 expression was associated with TP53 mutation status. **(D)** Promoter methylation level in distinct sample types from TCGA samples, suggesting that TOMM34 expression was associated with sample type. **(E)** Promoter methylation levels in distinct histological subtypes from TCGA samples, suggesting that TOMM34 expression was associated with histological subtype. **(F)** Protein expression of TOMM34 in distinct sample types, revealing that proteomic expression of TOMM34 was upregulated in tumor tissues compared with normal tissues. **(G)** Protein expression of TOMM34 at different ages. **(H)** Protein expression of TOMM34 in distinct clinical stages. **(I)** Survival analysis of TOMM34 in overall survival, indicating that upregulation of TOMM34 was remarkably related to poor prognosis in overall survival. ***p<0.001, **0.001≤p<0.01, *0.01≤p<0.05.

By using the UALCAN online tool, we investigated the correlations between TOMM34 expression and clinical traits of colon cancer in the TCGA and CPTAC modules. The results demonstrated that the gene expression of TOMM34 was dramatically associated with histological subtype and TP53 mutation status (**Figures 2A, B**). Analysis of promoter methylation showed that there were apparent differences in TP53 mutation, sample types, and histological subtypes (**Figures 2C-E**). Using the CPTAC module of UALCAN, analysis of proteomic expression revealed that proteomic expression of TOMM34 was upregulated in tumor tissues compared with normal tissues (**Figure 2F**). As shown in **Figures 2G, H**, further analysis of proteomic expression revealed that there were significant differences in age (no than 40 years old versus older) and clinical stage (stage II versus stage IV). Survival analysis indicated that upregulation of TOMM34 was remarkably related to poor prognosis in overall survival based on the TCGA-COAD cohort (**Figure 2I**).

### Identification of TOMM34 interaction genes and proteins and functional analysis

**Figure 3:**
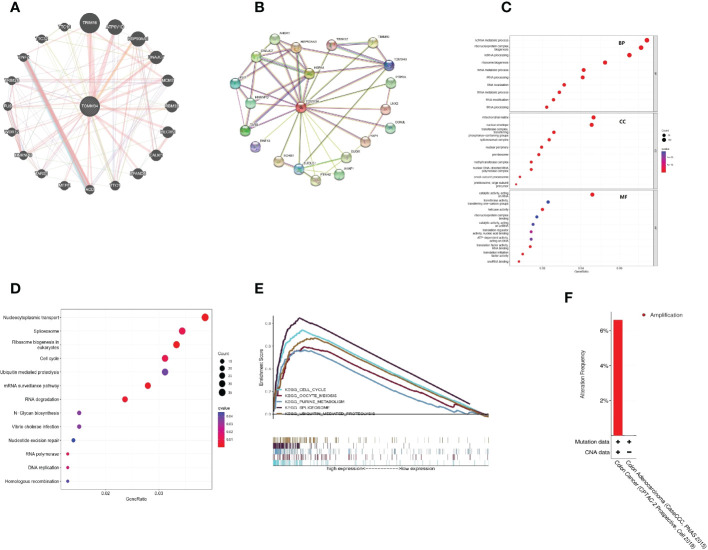
Interaction analysis of TOMM34-related genes and proteins and functional analyses. **(A)** Interaction analysis of related genes. **(B)** Interaction analysis of related proteins. **(C)** GO enrichment analysis. **(D)** KEGG pathway analysis. **(E)** GSEA based on KEGG analysis. **(F)** Genomic alteration type of TOMM34, revealing that the genomic alteration type of TOMM34 was amplification.

By using the GeneMANIA database, a gene−gene interaction network was constructed to show the interaction between TOMM34 and related genes (**Figure 3A**). The results indicated that the TOMM34 gene was closely related to the 20 most related genes, including TIM16, ATP6V1D, HSP90AA1 and DNAJC7. Additionally, we used the STRING database to construct the protein−protein interaction network to evaluate the relationship between TOMM34 and related proteins (**Figure 3B**). There were 21 nodes and 48 edges, including HSP90AA1, HSPA4 and DNAJC7. GO enrichment and KEGG pathway analyses were performed to evaluate the biological functions related to TOMM34. As shown in **Figure 3C**, TOMM34 was dramatically related to ncRNA metabolic processes, ribonucleoprotein complex biogenesis, ncRNA processing, ribosome biogenesis, and rRNA metabolic processing in terms of GO-BP. In terms of GO-CC, TOMM34 was enriched in the mitochondrial matrix, nuclear envelope, transferase complex, transferring phosphorus-containing groups, spliceosomal complex and unclear periphery. GO-MF enrichment analysis revealed that TOMM34 was significantly associated with catalytic activity on RNA, transferase activity, transferring one-carbon groups, helicase activity, ribonucleoprotein complex binding, and catalytic activity on tRNA. In addition, KEGG pathway analysis indicated that TOMM34 was enriched in nucleocytoplasmic transport, spliceosome, ribosome biogenesis in eukaryotes, cell cycle, and ubiquitin-mediated proteolysis (**Figure 3D**).

Moreover, we conducted multi-GSEA to further investigate the molecular mechanisms of TOMM34 in colon cancer. Among the KEGG terms, GSEA revealed that the top five signaling pathways were enriched in the cell cycle, oocyte meiosis, purine metabolism, spliceosome and ubiquitin-mediated proteolysis (**Figure 3E**). These findings strongly suggest that TOMM34 is implicated in the regulation of proliferation in colon cancer.

### Correlation between TOMM34 and gene mutation and microsatellite instability (MSI)

**Figure 4:**
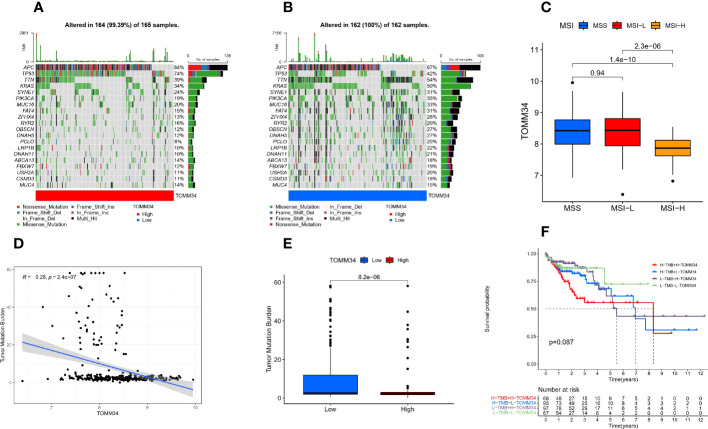
Somatic mutation analysis characteristics. **(A)** Distribution of the top 20 mutated genes in the high TOMM34 group. **(B)** Distribution of the top 20 mutated genes in the low TOMM34 group. **(C)** Correlation between TOMM34 and microsatellite instability (MSI), indicating that TOMM34 was negatively associated with high MSI. **(D)** Correlation between TOMM34 and tumor mutation burden (TMB), indicating that TOMM34 was negatively associated with TMB. **(E)** Boxplot showing the tumor mutation burden in the low and high TOMM34 groups. **(F)** Survival analysis of TOMM34 combined with tumor mutation burden, showing that patients with high TMB and high TOMM34 presented worse prognosis. MSI, microsatellite instability; MSS, microsatellite stability; MSI-L, low microsatellite instability; MSI-H. high microsatellite instability; H-TMB, high tumor mutation burden; L-TMB, low tumor mutation burden; H-TOMM34, high expression of TOMM34; L-TOMM34, low expression of TOMM34.

First, we found that the genomic alteration type of TOMM34 was amplification (**Figure 3F**). Between the high and low TOMM34 groups, we compared somatic tumor mutation profiles to investigate the relationship between TOMM34 and TMB. The top 20 mutated genes were chosen to compare the differences in mutation frequency. APC and TP53 were frequently mutated in the high TOMM34 group (**Figure 4A**). However, the remaining 18 genes, including TTN, KARAS and PIK3CA, were frequently mutated in the low TOMM34 group (**Figure 4B**). We also evaluated the relationship between TOMM34 and MSI and TMB (**Figures 4C-E**). The results revealed that TOMM34 was negatively associated with TMB and high MSI. Survival analysis of TOMM34 combined with TMB showed that patients with high TMB and high TOMM34 presented a shorter survival prognosis than those with low TMB and low TOMM34 (**Figure 4F**).

### Correlation analysis between TOMM34 and immune cell infiltration in the tumor microenvironment

**Figure 5:**
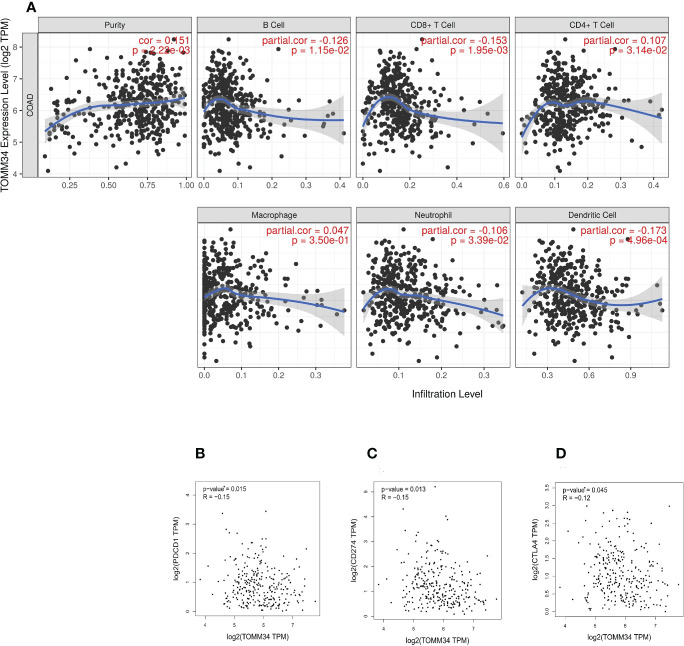
Correlation between TOMM34 expression and immune cell infiltration level. **(A)** The correlation between TOMM34 expression and distinct immune cell infiltration by using the TIMER database, revealing that TOMM34 expression was positively associated with the infiltration of CD4+ T cells and was not correlated with macrophages in colon cancer. **(B)** Correlations between TOMM34 expression and PD-1, indicating that TOMM34 was negatively associated with PD-1. **(C)** Correlations between TOMM34 expression and PD-L1, indicating that TOMM34 was negatively associated with PD-L1. **(D)** Correlations between TOMM34 expression and CTLA-4, indicating that TOMM34 was negatively associated with CTLA-4.

The TIMER online tool was utilized to perform correlation analysis between TOMM34 expression and infiltration of six types of immune cells in colon cancer, including B cells, CD8+ T cells, CD4+ T cells, macrophages, neutrophils and dendritic cells. Our results demonstrated that there were significantly negative relationships between TOMM34 expression and the infiltration of B cells, CD8+ T cells, neutrophils and dendritic cells. TOMM34 expression was positively associated with the infiltration of CD4+ T cells and was not correlated with macrophages in colon cancer (**Figure 5A**). Additionally, we evaluated the correlation between TOMM34 expression and immune checkpoints, such as PD-1, PD-L1 and CTLA-4. On the basis of the GEPIA database, TOMM34 was negatively associated with PD-1, PD-L1 and CTLA-4 (**Figures 5B-D**).

**Figure 6:**
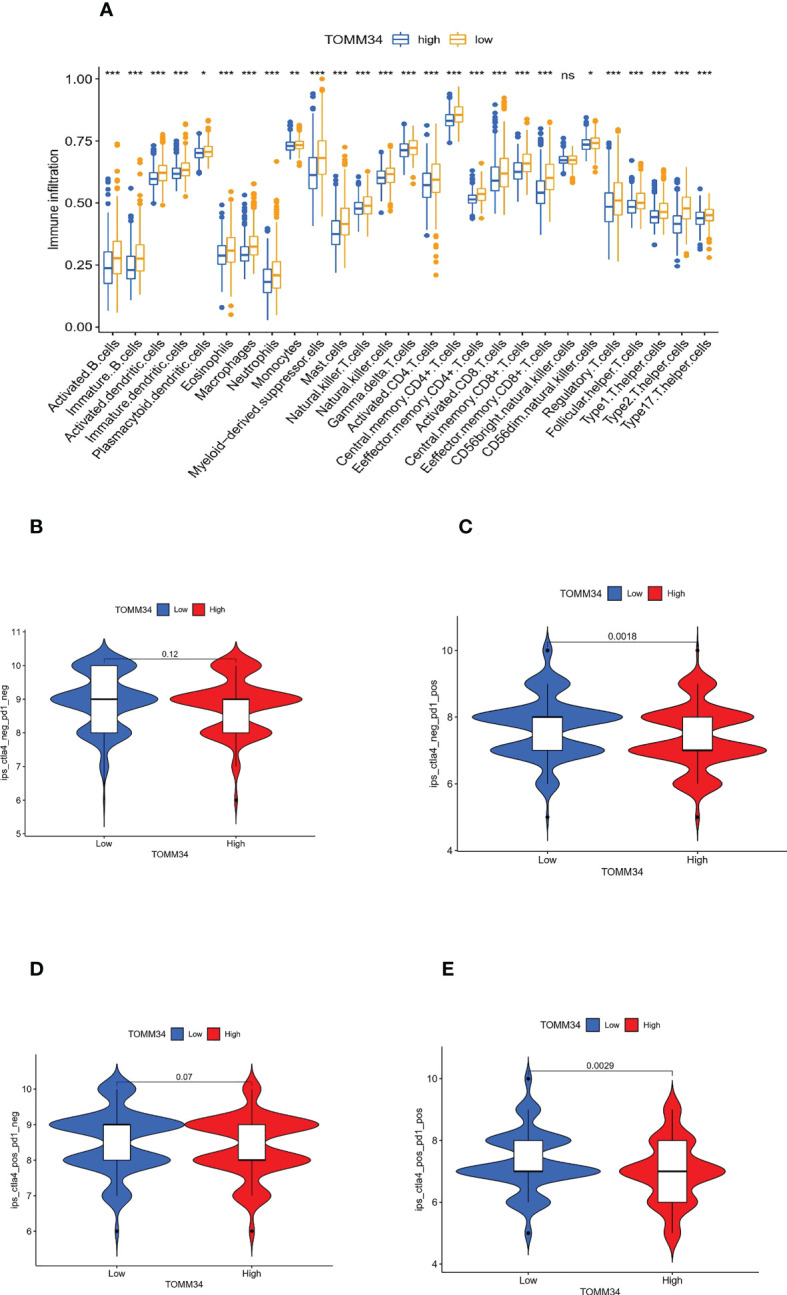
Correlation between TOMM34 expression and immune cell infiltration level. **(A)** Immune cell infiltration levels in distinct TOMM34 groups, showing that 26 types of immune cells except CD56 bright natural killer cells were elevated in the low TOMM34 group compared with the high TOMM34 group. **(B-E)** Violin plot of IPS among ips_ctla4_neg_pd1_neg (CTLA-4 negative response and PD-1 negative response), ips_ctla4_neg_pd1_pos (CTLA-4 negative response and PD-1 positive response), ips_ctla4_pos_pd1_neg and ips_ctla4_pos_pd1_pos in the respective TOMM34 groups, showing that patients in the low TOMM34 group displayed significant clinical advantages for anti-PD-1 immunotherapy. ***p<0.001, **0.001≤p<0.01, *0.01≤p<0.05, ns, p>0.05.

Moreover, ssGSEA was performed to further evaluate the correlation between TOMM34 expression and immune cell infiltration in the tumor microenvironment (TME). Except for CD56 bright natural killer cells, the remaining 26 types of immune cells were elevated in the low TOMM34 group compared with the high TOMM34 group (**Figure 6A**).

Additionally, we employed the TIMER database to evaluate the relationship between TOMM34 expression and distinct immune signatures in colon cancer for a comprehensive understanding of TOMM34 crosstalk with the immune response. Based on a validated gene list (27), immune cells were identified (**Table 1**). After adjusting for tumor purity, TOMM34 was remarkably related to most immune signatures in a variety of immune cells in colon cancer. We also investigated the correlation between various functional T cells and TOMM34 expression. As shown in **Table 2**, we found that TOMM34 was dramatically related to 29 of 38 T-cell markers in colon cancer and associated with 18 of 38 T-cell markers in colon cancer after adjusting for tumor purity.

**Table 1:** Correlation analysis between TOMM34 and gene markers of immune cells in TIMER.

Description	Gene markers	None		Purity	
		Correlation	*P* value	Correlation	*P* value
T cell (general)	CD2	-0.199	***	-0.153	**
	CD3D	-0.236	***	-0.209	***
	CD3E	-0.209	***	-0.175	***
CD8+ T cell	CD8A	-0.208	***	-0.177	***
	CD8B	-0.053	0.256	-0.029	0.566
B cell	CD19	-0.092	0.05	-0.036	0.476
	CD79A	-0.113	*	-0.053	0.287
Monocyte	CD86	-0.179	***	-0.13	**
	CSF1R	-0.179	***	-0.152	**
Tumor-associated macrophage	CCL2	-0.049	0.294	-0.015	0.756
	CD68	-0.171	***	-0.136	**
M1 macrophage	IRF5	0.108	*	0.107	*
	PTGS2	-0.178	***	-0.164	***
	NOS2	-0.176	***	-0.155	**
M2 macrophage	CD163	-0.18	***	-0.147	**
	VSIG4	-0.208	***	-0.185	***
	MS4A4A	-0.21	***	-0.176	***
Neutrophils	CCR7	-0.104	*	-0.075	0.132
	ITGAM	-0.225	***	-0.196	***
	CEACAM8	0.02	0.67	-0.009	0.856
Natural killer cell	KIR2DL1	-0.207	***	-0.205	***
	KIR2DL3	-0.189	***	-0.143	**
	KIR2DL4	-0.271	***	-0.238	***
	KIR3DL1	-0.172	***	-0.133	**
	KIR3DL2	-0.211	***	-0.188	***
	KIR3DL3	-0.132	**	-0.11	*
	KIR2DS4	-0.122	**	-0.103	*
Dendritic cell	CD1C	-0.073	0.12	-0.04	0.419
	HLA-DPB1	-0.29	***	-0.257	***
	HLA-DRA	-0.255	***	-0.221	***
	HLA-DPA1	-0.218	***	-0.178	***
	ITGAX	-0.147	**	-0.113	*
	NRP1	-0.07	0.134	-0.017	0.731

*0.01≤P<0.05, **0.001≤P<0.01, ***P<0.001

**Table 2:** Correlation analysis between TOMM34 and gene markers of distinct types of T cells in TIMER.

Description	Gene markers	None		Purity	
		Correlation	*P* value	Correlation	*P* value
Th1	TBX21	-0.16	***	-0.112	*
	TNF	-0.094	*	-0.06	0.228
	STAT1	-0.028	0.557	0.001	0.979
	STAT4	-0.206	***	-0.182	***
	IFNG	-0.098	*	-0.076	0.129
Th1-like	HAVCR2	-0.197	***	-0.164	***
	IFNG	-0.098	*	-0.076	0.129
	CD4	-0.141	**	-0.102	*
	BHLHE40	-0.052	0.263	-0.006	0.909
	CXCR3	0.268	***	0.281	***
Th2	STAT6	0.093	*	0.076	0.126
	STAT5A	-0.041	0.383	-0.02	0.692
Treg	FOXP3	-0.077	*	-0.03	0.301
	CCR8	-0.025	0.591	0.009	0.796
	TGFB1	-0.197	***	-0.171	***
Resting Treg	FOXP3	-0.077	*	-0.03	.0301
	IL2RA	-0.16	***	-0.109	*
Effector Treg T cell	FOXP3	-0.077	*	-0.03	0.301
	CCR8	-0.025	0.591	0.009	0.796
	TNFRSF9	-0.123	**	-0.084	*
Effector T cell	CX3CR1	-0.013	.0778	-0.004	0.940
	FGFBP2	-0.253	***	-0.26	***
	FCGR3A	-0.171	***	-0.134	**
Naïve T cell	CCR7	-0.104	*	-0.075	0.132
	SELL	-0.11	*	-0.066	0.183
Effector memory T cell	DUSP4	-0.542	***	-0.535	***
	GZMK	-0.16	***	-0.11	*
	GZMA	-0.245	***	-0.195	***
Resident memory T cell	CD69	-0.256	***	-0.23	***
	CXCR6	-0.176	***	-0.143	**
	MYADM	-0.049	0.291	-0.024	0.624
General T cell	CCR7	-0.104	*	-0.075	0.132
Memory T cell	SELL	-0.11	*	-0.066	0.183
	IL7R	-0.073	0.118	-0.032	0.522
Exhausted T cell	LAYN	-0.043	0.352	-0.001	0.978
	HAVCR2	-0.197	***	-0.164	***
	LAG3	-0.208	***	-0.179	***
	CXCL13	-0.168	***	-0.117	*

*0.01≤P<0.05, **0.001≤P<0.01, ***P<0.001

To evaluate the role of TOMM34 in predicting immunotherapeutic benefits, we utilized The Cancer Immunome Atlas (ICIA) to assess the immune response. We further predicted the value of the risk score for immune checkpoint blockade (ICB). The IPS results of TCGA-PRAD were downloaded from the TCIA website. PD-1 and CTLA-4 were included in immunophenoscore (IPS) analysis, and classified into four parts: ips_ctla4_neg_pd1_neg (CTLA-4 negative response and PD-1 negative response), ips_ctla4_neg_pd1_pos (CTLA-4 negative response and PD-1 positive response), ips_ctla4_pos_pd1_neg and ips_ctla4_pos_pd1_pos. In the two parts of the positive response of PD-1, the average IPS in the low TOMM34 group was significantly higher than that in the high TOMM34 group, showing that patients in the low TOMM34 group displayed significant clinical advantages for anti-PD-1 immunotherapy (**Figures 6B-E**). Similar to the abovementioned results, these results indirectly confirmed that TOMM34 has a pivotal effect on mediating the immune response in colon cancer.

### Immunohistochemical analysis of tumor tissue and normal tissue in 206 samples

**Figure 7:**
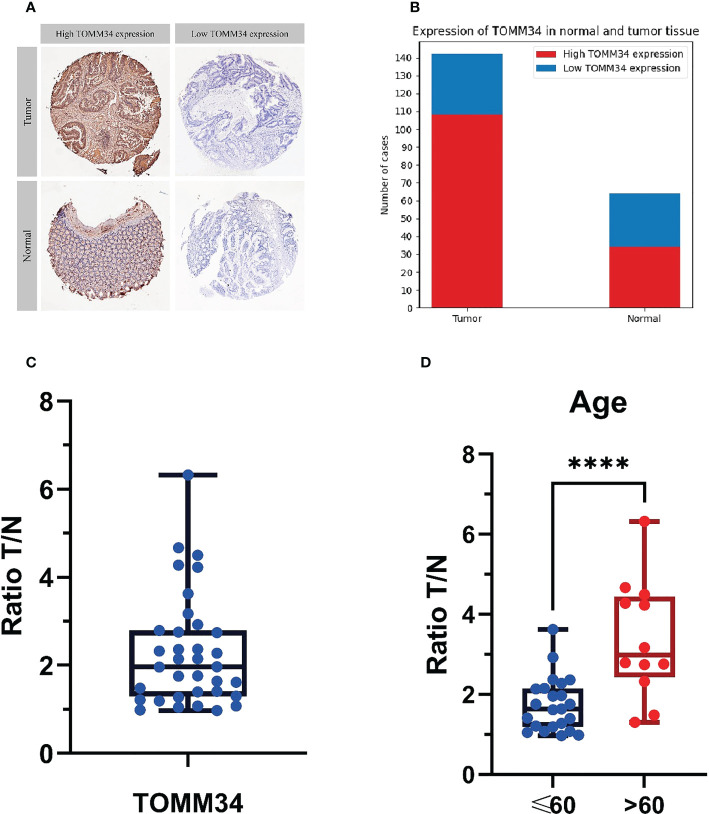
Immunohistochemical analysis of 206 samples and mass spectrometry analysis of tumor tissue and normal tissue in 35 patients. **(A)** The judgment standard for immunohistochemistry of TOMM34, in which cells stained in dark brown are defined as high expression, and unstained or light yellow are defined as low expression. **(B)** Qualitative analysis of the expression levels of TOMM34 in normal and tumor tissues by immunohistochemistry indicated that the expression levels of TOMM34 in tumor tissues were significantly higher than those in normal tissues. **(C)** Quantitative analysis of the expression of TOMM34 in tumor tissues and adjacent normal tissues of 35 colon cancer patients by mass spectrometry, showing that the expression of TOMM34 in tumor tissue was significantly higher than that in adjacent normal tissue. **(D)** The ratio tumor/normal (T/N) of patients under 60 years old and patients over 60 years old. ****p<0.0001.

We performed immunohistochemical analysis on 206 samples obtained from screening, of which 142 were tumor tissues and 64 were normal tissues (**Figures 7A, B**).

Among all samples, 142 cases had high expression of TOMM34, while 64 cases had low expression. Among the 142 tumor tissue samples, 108 (76.06%) had high expression of TOMM34, and 34 (23.94%) had low expression. Among the 64 normal tissue samples, 34 (53.13%) had high expression of TOMM34, and 30 (46.87%) had low expression. Statistical analysis (chi-square test) showed that the difference in TOMM34 expression between tumor tissue and normal tissue was statistically significant (χ²=10.832, *P*=0.001).

### LC−MS/MS analysis of tumor tissue and normal tissue in 35 patients

We qualitatively analyzed the expression of TOMM34 in tumor tissues and adjacent normal tissues of 35 colon cancer patients by LC−MS/MS analysis (**Figures 7C, D**).

LC−MS/MS data showed that the expression of TOMM34 was significantly different in tumor tissue and adjacent normal tissue [false discovery rate (FDR) < 0.05, median ratio tumor/normal (T/N) = 2.106)], indicating that TOMM34 expression was upregulated in tumor tissues. The T test showed that the T/N ratio of patients under 60 years old was significantly lower than that of patients over 60 years old (*P*=0.0021). However, the T/N ratio was not significantly different among patients with different sexes, different stages, and different lesion locations.

## Discussion

Colon cancer represents the fourth most common lethal malignancy globally (28) and has both strong genetic risk factors and environmental relevance. An accumulation of genetic alterations allows the transformation of the normal colonic epithelium to a precancerous lesion and ultimately to invasive carcinoma (29). Despite advances in early screening and combined therapies, the prognosis of colon cancer patients remains unsatisfactory because of tumor recurrence and metastasis. It has been reported that the long-term survival rate is only approximately 50% (30). Thus, it is urgent to comprehensively explore the underlying mechanisms of colon cancer progression. Accumulating evidence has confirmed that increased activity of the chaperones Hsp70 and Hsp90 is a hallmark of solid tumors. TOMM34 is a cochaperone of both Hsp70 and Hsp90, which has been found to contribute to tumorigenesis and progression of hepatocellular carcinoma, colorectal cancer and breast cancer. However, the underlying function of TOMM34 in colon cancer remains to be illustrated.

In the present study, we found that TOMM34 expression was elevated in colon cancer tissues compared with normal tissues by means of integrated bioinformatics analysis. These results were validated at the gene and protein levels using the UALCAN database. Consistent with previous studies (24, 25, 31), these results confirmed that upregulation of TOMM34 expression was significantly associated with unfavorable prognosis in colon cancer and indicated that TOMM34 may serve as an oncogene by facilitating tumor progression and immunosuppression. The overexpression of TOMM34 may be a crucial factor in the occurrence and progression of colon cancer because previous studies have also shown that TOMM34 plays an important role in tumor cell growth (18). Subsequently, we analyzed the correlation between TOMM34 expression and immune cell infiltration using the TIMER database. High TOMM34 was negatively related to divergent types of immune cells, including B cells, CD8+ T cells, neutrophils and dendritic cells, and negatively associated with PD-1, PD-L1 and CTLA-4. Furthermore, survival analysis showed that colon cancer patients with high TOMM34 expression exhibited a significantly poorer prognosis. By using the cBioPortal database, we found that 6.7% of colon cancer patients have gene alterations in TOMM34, of which gene amplification is the most common alteration. Our findings substantiated that TOMM34 is a potential prognostic biomarker and promising therapeutic target against colon cancer.

To our knowledge, the correlation between TOMM34 and immune cell infiltration in colon cancer has not been evaluated. We utilized GO enrichment and KEGG pathway analyses to investigate the correlation between immune cell infiltration and TOMM34 in colon cancer. Our results revealed that TOMM34 is implicated in numerous pathways, especially the immune system, in colon cancer. These findings confirmed that high TOMM34 expression is negatively associated with the increased infiltration of B cells, CD8+ T cells, neutrophils and dendritic cells in colon cancer. However, high TOMM34 expression was positively associated with increased CD4+ T cell infiltration. This mechanism has not been elucidated, but previous studies have shown that the expression of TOMM34 is upregulated when CD4+ T cells are activated, while its expression is not significantly upregulated when CD8+ T cells are activated (32). Additionally, the relationships between TOMM34 and distinct immune cell signatures have been found to be significant. Overexpression of TOMM34 was also negatively related to the expression of PD-1, PD-L1 and CTLA-4. More notably, correlations between the levels of immune cell infiltration of tumors in colon cancer and prognosis have been investigated in previous studies, which have shown that the degree of infiltration of various immune cells, including CD8+ T cells, is significantly associated with better prognosis, while the degree of infiltration of some CD4+ T cells (such as Th17 cells) is associated with poorer prognosis (33, 34). Therefore, TOMM34 was remarkably associated with shorter survival in colon cancer partially *via* immune cell infiltration. The present study indicated that TOMM34 could serve as a potential immunotherapeutic target against colon cancer, especially in patients with advanced-stage disease or metastasis. Nevertheless, the underlying roles of TOMM34 in the tumor microenvironment still need to be further investigated.

In 1908, Paul Ehrlich proposed that the immune system has the capacity to suppress tumorigenesis and thus plays an important role in protection against tumor progression (35, 36). Recently, immunotherapy has become one of the most prominent weapons against cancer, especially for patients with advanced stages or metastatic disease. The major obstacle to immunotherapeutic vaccination strategies is the identification of tumor-associated antigens (TAAs). Recent advances in checkpoint blockade inhibitors have promoted the development of cancer immunotherapy (37). The major issue with immunotherapy is specificity, as only a large number of patients experience a durable clinical benefit (37, 38). The relationship between TOMM34 and immune checkpoints has not been elucidated. Previous studies have shown that the upregulation of TOMM34 and several other genes may regulate the interferon signaling pathway by affecting mitochondrial activity (32), and the interferon signaling pathway has been proven to be associated with resistance to immune checkpoint blockade (39). A clinical trial of the peptide vaccines RNF43 and TOMM34 combined with chemotherapy for stage III colorectal cancer revealed that patients with a positive CTL response showed good 3-year relapse-free survival regardless of HLA-A*2402 status (22). This evidence confirmed that TOMM34 is associated with immune cell infiltration in colon cancer.

Although the present study facilitates our comprehensive understanding of the correlation between TOMM34 and colon cancer, there are some limitations that should be noted. First, we explored the relationship between TOMM34 and immune cell infiltration at the gene level, which has not been experimentally validated, especially at the protein level. Additionally, we only investigated the relationship in colon cancer, and that in other types of cancer needs to be further evaluated. Moreover, the molecular mechanisms of TOMM34 in immune cell infiltration, escape, tumorigenesis and progression were not investigated in the present study.

Overall, our findings revealed that TOMM34 is a candidate prognostic biomarker and promising immunotherapeutic target against colon cancer. Moreover, the present study investigated the underlying evidence that TOMM34 regulates immune cell infiltration in colon cancer. Therefore, our study will provide insights into not only the correlation between TOMM34 and colon cancer but also the mechanism of immunotherapy.

## Data availability statement

The original contributions presented in the study are included in the article/**Supplementary Material**. Further inquiries can be directed to the corresponding author.

## Author contributions

ZL, LL and XW contributed to the design of this work. ZL, HY and JL downloaded these data from the online database and analyzed the data. ZL and HY wrote this manuscript. XW and LL supervised and revised this manuscript. All authors contributed to the article and approved the submitted version.
